# Hormesis in the Assessment of Toxicity Assessment by Luminescent Bacterial Methods

**DOI:** 10.3390/toxics12080596

**Published:** 2024-08-17

**Authors:** Haoyu Si, Guoquan Zhou, Yu Luo, Zhuoxuan Wang, Xuejun Pan, Guohua Dao

**Affiliations:** 1 Faculty of Environmental Science and Engineering, Kunming University of Science and Technology, Kunming 650500, China; 17616512715@163.com (H.S.); zhou94709@163.com (G.Z.); foreverkerry@126.com (Y.L.); wangzhuoxuanzml@163.com (Z.W.); xjpan@kust.edu.cn (X.P.); 2 Yunnan Academy of Ecological and Environmental Science, Yunnan Key Laboratory of Pollution Process and Control of Plateau Lake-Watersheds, Kunming 650034, China

**Keywords:** luminescent bacterial, hormesis, toxicity assessment

## Abstract

The threat posed by water pollutants to aquatic ecosystems and human health cannot be overlooked, and the assessment of the toxicity of these contaminants is paramount to understanding their risks and formulating effective control measures. Luminescent bacteria-based assays, as a vital tool in evaluating contaminant toxicity, encounter a challenge in ensuring accuracy due to the phenomenon of “Hormesis” exhibited by pollutants towards biological entities, which may skew toxicity assessments. This study elucidated the specific effects of pollutants on luminescent bacteria at different concentrations, used modeling to characterize the effects and predict their toxicity trends, and explored the applicable concentration ranges for different pollutants. Research revealed that six typical pollutants, namely PAHs, endocrine disruptors, antibiotics, pesticides, heavy metals, and phytosensory substances, could promote the luminescence intensity of luminescent bacteria at low concentrations, and the promotional effect increased and then decreased. However, when the concentration of the substances reached a certain threshold, the effect changed from promotional to inhibitory, and the rate of inhibition was directly proportional to the concentration. The EC50 values of six types of substances to luminescent bacteria is as follows: endocrine disruptors > pesticides > antibiotics > heavy metals > polycyclic aromatic hydrocarbons > chemosensory agents. The effect curves were further fitted using the model to analyze the maximum point of the promotion of luminescence intensity by different substances, the threshold concentration, and the tolerance of luminescent bacteria to different substances. The maximum promotion of bacterial luminescence intensity was 29% for Bisphenol A at 0.005 mg/L and the minimum threshold concentration of chromium was 0.004 mg/L, and the maximum bacterial tolerance to erythromycin is 6.74. In addition, most of the current environmental concentrations had a positive effect on luminescent bacteria and may still be in the range of concentrations that promote luminescence as the substances continue to accumulate. These findings will enhance the accuracy and comprehensiveness of toxicity assessments, thereby facilitating more informed and effective decision-making in the realms of environmental protection and pollution management.

## 1. Introduction

With the rapid acceleration of industrialization and urbanization, the issue of water pollution has garnered considerable attention in environmental research circles, owing to its increasingly severe consequences on aquatic ecosystems and human health. A diverse array of pollutants, encompassing polycyclic aromatic hydrocarbons (PAHs) [1], endocrine disruptors [2,3], antibiotics [4], pesticides [5], heavy metals [6,7], and phytosensory substances (PSMs) [8], have been detected in water bodies. These contaminants infiltrate aquatic environments primarily through anthropogenic activities such as industrial discharges and agricultural runoff. Although their initial concentrations in the water may seem low [9], their prolonged accumulation can exert progressively detrimental effects on aquatic ecosystems, underscoring the urgency for effective water pollution control measures.

The presence of pollutants in aquatic systems poses profound threats to both aquatic ecosystems and human health. Their toxicity disrupts the delicate equilibrium of the aquatic food chain, causing abnormalities in fish populations, fostering excessive algal blooms that can deplete oxygen levels, and degrading water quality to unsustainable levels. Moreover, these pollutants possess the ability to bioaccumulate within the food chain, amplifying their concentrations as they ascend [10], ultimately reaching humans through the consumption of contaminated seafood, drinking water, or even through dermal absorption. This poses a dire risk to human health, potentially leading to a wide range of health issues, including cancer, reproductive disorders, and endocrine system imbalances [11,12]. Hence, the assessment of the toxicity of these pollutants is paramount in devising effective pollution control strategies. A deep understanding of the nature, extent, and dynamics of the risks posed by these pollutants can be achieved through the application of rigorous scientific assessment methods. This knowledge base facilitates the development of targeted interventions to mitigate their harmful effects and restore the resilience of our aquatic environment. In addition, it informs the development of policies and regulations aimed at preventing future pollution, protecting the health of aquatic organisms, and preserving the integrity of our water resources.

In the ever-evolving landscape of environmental monitoring, a diverse array of techniques have emerged to assess the toxicity of pollutants, spanning from conventional chemical analysis to cutting-edge biological assays [13,14]. Among them, luminescent bacterial testing is a promising tool that is widely used for toxicity assessment of toxic substances [15]. This assay harnesses the innate bioluminescent properties of specific bacterial strains, where pollutants can disrupt the intricate enzymatic pathways responsible for light emission or impede their energy metabolism, resulting in a diminished luminescence output [16]. The decline in luminescence intensity serves as an indicative marker for the presence, concentration, and toxicity of contaminants, endowing the method with remarkable sensitivity and swift responsiveness. Although luminescent bacteriological assays stand out in detecting high contaminant concentrations, owing to their sensitivity and ease of use, it is imperative to acknowledge their constraints when dealing with low exposure levels. Specifically, low concentrations of contaminants that paradoxically stimulate bacterial luminescence can introduce inaccuracies in the results, highlighting the need for caution in interpretation [17]. The latest study showed that the bacteria employed in the luminescent assay not only exhibit remarkable sensitivity to pollutants at high concentrations, but also possess intriguing adaptive mechanisms that may affect their response patterns under varying environmental stressors [18]. Specifically, the study revealed that certain strains of luminescent bacteria can undergo physiological changes in response to low concentrations of pollutants, potentially altering their bioluminescent properties in ways that complicate straightforward toxicity assessments. These adaptive mechanisms, such as changes in gene expression or metabolic pathways, suggest that the bacteria’s response to pollutants is more nuanced than previously understood and underscores the need for further research to refine the luminescent bacteria assay for broader applicability and improved accuracy across a wider range of contaminant concentrations [19]. This phenomenon underscores the importance of considering the concentration range when interpreting results from the luminescent bacteria test, as it may yield inaccurate toxicity assessments under certain conditions.

Therefore, while the luminescent bacteria assay retains its position as a valuable and cost-effective tool for environmental monitoring and pollution control, particularly for the detection of high-concentration pollutants, its application necessitates a nuanced appreciation of its inherent limitations and an acknowledgment of the potential for hormetic effects—where low concentrations may stimulate rather than inhibit bacterial bioluminescence—at lower pollutant concentrations. By integrating this nuanced understanding into monitoring protocols, we can elevate the accuracy and comprehensiveness of toxicity assessments, thereby fostering more informed and effective decision-making in the realms of environmental protection and pollution management. The present study was designed to delve into the dynamics of toxicity exhibited by diverse substances towards luminescent bacteria at environmentally relevant concentrations, as well as to examine these effects during their accumulation. Furthermore, the study aimed to establish the concentration range within which the LB method can be reliably employed for determining the toxicity of various substances, contributing to the refinement and optimization of this crucial tool in environmental toxicology.

## 2. Materials and Methods

### 2.1. Strains and Chemicals

The freeze-dried powder luminescent bacteria of the strain *Photobaclerium phosphortum* T3 was provided by the Microbiology Department, Nanjing Institute of Soil Research, Chinese Academy of Sciences (Nanjing, China).

Six categories of typical pollutants were selected as target substances, each including two pollutants. PAHs: Naphthalene and Benzene; endocrine disruptors: Dichlorophenol and Bisphenol A; antibiotics: Kanamycin sulfate and Erythromycin; pesticides: Trichlorfon and Glyphosate; heavy metals: Chromium and Lead; and allelochemicals: Gallic acid and Nonanoic acid. All 12 substances were purchased from Shanghai Macklin Biochemical Co., Ltd. (Shanghai, China).

### 2.2. Resuscitation of Luminescent Bacteria

In the determination of 48 h before taking the preserved strains, in the fresh slant, to receive the first generation of slant, 20 °C culture 24 h is immediately transferred to the second generation of slant, 20 °C culture 12 h, and then receives the third generation of slant, 20 °C culture 12 h, and prepared for use. Take the third generation of slant strains inoculated in a 250 mL triangular flask containing 50 mL culture solution, (20 °C, 180 r/min under culture for 14 h). The culture solution is then diluted until the initial luminescence was not less than 800 mV and set aside in an ice bath [20].

### 2.3. Acute Toxicity Assays for Chemicals

Considering the toxicity of the contaminants and the varying sensitivities of luminescent bacteria, the concentrations of the target contaminants were established by pre-experimentation. On the other hand, concentration settings for pollutants include low concentrations in their natural waters, gradual increases in concentrations in natural waters due to production and consumption, and relatively high concentrations that may be reached by accumulating pollutants in natural waters over a considerable period of time. All samples were diluted with 3% NaCl solution at a temperature of 20 °C. The pH of solutions was adjusted to 7.00 ± 0.05 by 0.1 mol/L NaOH or HCl solution. All solutions were prepared using ultra-pure water from a Milli-Q system (Millipore, Bedford, MA, USA). Conducting the assays, the solution of activated *P. phosphoreum* T3 was stored in an ice water bath; 10 μL of the solution was put into the tubes containing 2 mL of compound solution or 3% NaCl solution (as control) [21]. After exposure for 15 min, the luminous intensity of *P. phosphoreum* T3 was recorded by using the DXY-2 Biological Toxicity Tester (Institute of Soil Science, Chinese Academy of Sciences, China). The inhibition rate is calculated by Equation (1). The toxicity data with the inhibition were used to fit a straight line, and therefore to calculate the EC_50_ value. For each concentration, a set of three biological replicates were performed and each sample was assayed three times using the same technical method.

(1) $$Inhibition\;rate=\frac{L_{0}-L_{x}}{L_{0}}\times 100\%$$

### 2.4. Fitting the Dose–Response Model

The luminous intensity of *P. phosphoreum* T3 under the stress of chemicals could be calculated by Equation (2) [22]. The model postulates that chemicals possess opposing and unifying inhibitory and promotional properties due to their display of low promotional and high inhibitory characteristics. *S_x_* and *I_x_* represented the promotion and inhibition of luminescence intensity by the substance at dose *x*, respectively. *P*_0_ is the effect in the absence of substance dose stress, and in this study, *P*_0_ was 0. Therefore, Equation (3) is used in this study. When the facilitating property is dominant (*S_x_* > *I_x_*), the substance behaves as a facilitator of luminous intensity; when the inhibiting property is dominant (*S_x_* < *I_x_*), the substance behaves as an inhibitor of luminous intensity.

(2) $$P\left(x\right)=P_{0}+S_{x}-I_{x}$$

(3) $$P\left(x\right)=S_{x}-I_{x}\;$$

Additionally, the Monod equation is a commonly used kinetic model of biological reactions to describe the response to substrates in a given environment [23]. Therefore, the Monod equation is used as a sub-equation of the model Equation (4). Combining the two equations yields the new model Equation (5), which was further used to describe the “Hormesis” of chemicals on luminescent bacteria.

(4) $$\mu =\frac{\mu_{max}\times S}{K_{s}+S}$$

where *μ* is the specific growth rate, i.e., the rate of growth per unit of biomass (d^−1^); *μ*_*max*_ is the maximum specific growth rate (d^−1^); *K*_*S*_ is half-saturation constant, which is the substrate concentration when *μ* = *μ*_*max*_/2, *S* is the single limiting substrate concentration (mg·L^−1^).

(5) $$P\left(x\right)=\frac{S_{max}+x}{K_{s}+x}+\frac{I_{max}+x}{K_{i}+x}$$

where *S*_*max*_ is the maximum response of pure promoting organisms at saturating doses of trace elements; *I*_*max*_ is the maximum response of purely inhibited organisms at saturating doses of trace elements; *k*_*s*_ is a constant that promotes the response rate (steepness of the response curve); and *k*_*i*_ is a constant that inhibits the response rate (steepness of the response curve).

## 3. Results and Discussion

### 3.1. Toxicity of Different Substances to Luminescent Bacteria

**Figure 1:**
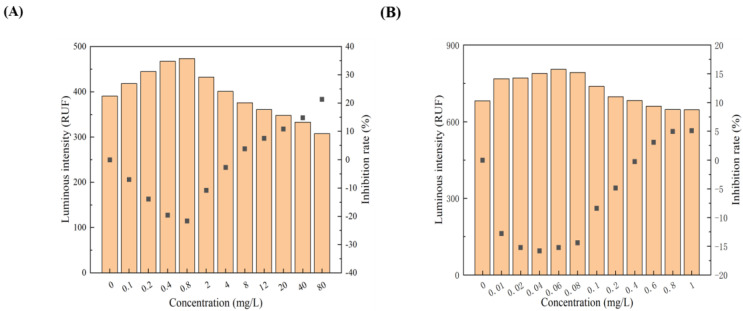
Effect of Naphthalene (**A**) and Benzene (**B**) on the luminous intensity of *P. phosphoreum* T3. The columns indicate the luminous intensity of *P. phosphoreum* T3 at a specific concentration, and the black dots indicate the inhibition rate of fluorescence intensity.

**Figure 2:**
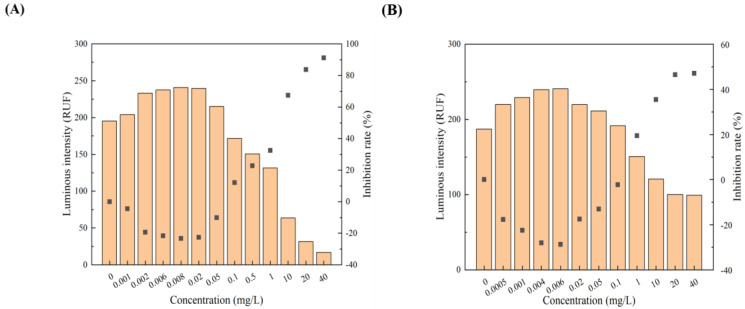
Effect of Dichlorophenol (**A**) and Bisphenol A (**B**) on the luminous intensity of *P. phosphoreum* T3. The columns indicate the luminous intensity of *P. phosphoreum* T3 at a specific concentration, and the black dots indicate the inhibition rate of fluorescence intensity.

**Figure 3:**
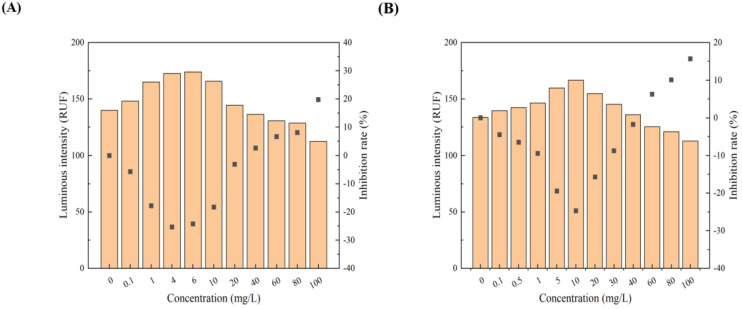
Effect of Kanamycin sulfate (**A**) and Erythromycin (**B**) on the luminous intensity of *P. phosphoreum* T3. The columns indicate the luminous intensity of *P. phosphoreum* T3 at a specific concentration, and the black dots indicate the inhibition rate of fluorescence intensity.

**Figure 4:**
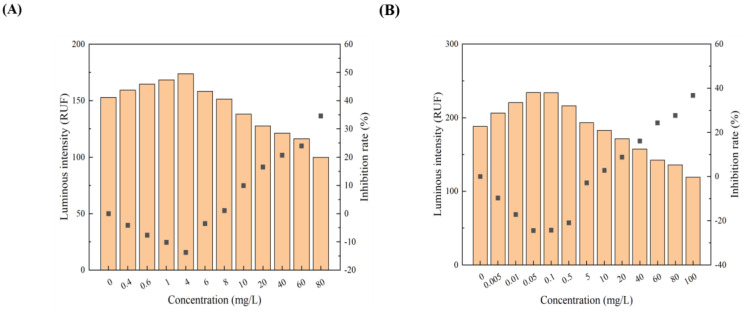
Effect of Trichlorfon (**A**) and Glyphosate (**B**) on the luminous intensity of *P. phosphoreum* T3. The columns indicate the luminous intensity of *P. phosphoreum* T3 at a specific concentration, and the black dots indicate the inhibition rate of fluorescence intensity.

**Figure 5:**
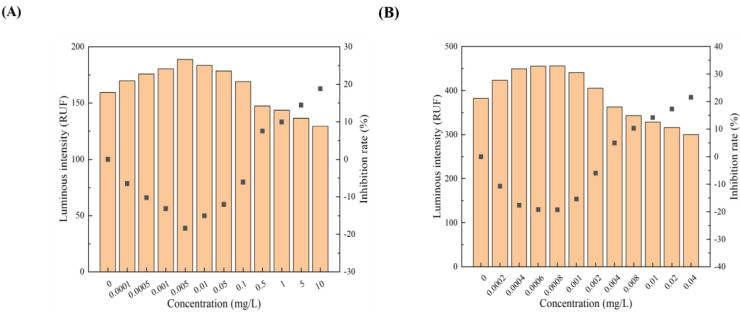
Effect of Chromium (**A**) and Lead (**B**) on the luminous intensity of *P. phosphoreum* T3. The columns indicate the luminous intensity of *P. phosphoreum* T3 at a specific concentration, and the black dots indicate the inhibition rate of fluorescence intensity.

**Figure 6:**
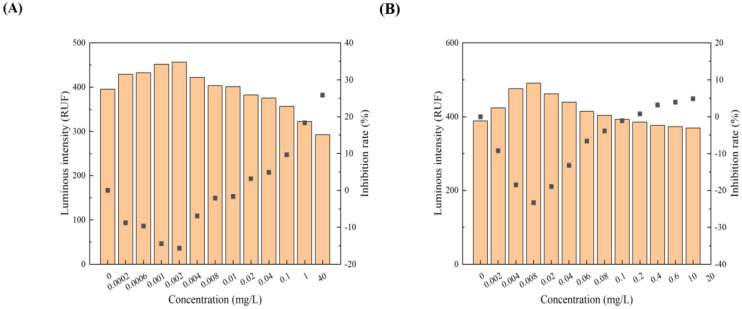
Effect of Gallic acid (**A**) and Nonanoic acid (**B**) on the luminous intensity of *P. phosphoreum* T3. The columns indicate the luminous intensity of *P. phosphoreum* T3 at a specific concentration, and the black dots indicate the inhibition rate of fluorescence intensity.

All 12 pollutants showed a tendency to promote the luminescence of *Photobaclerium phosphortum* T3 at low concentrations and inhibit it at high concentrations. For two PAHs, luminous intensity is enhanced within a concentration range of 0 to 4 mg/L Naphthalene and 0–0.4 mg/L Benzene (Figure 1). Two endocrine disruptors (Dichlorobiphenyl and Bisphenol a) promoted luminous intensity at concentrations of 0–0.05 and 0–0.1 mg/L (Figure 2). Kanamycin sulfate and Erythromycin enhanced the fluorescence intensity of luminescent bacteria at 0–40 mg/L and 0–20 mg/L, respectively (Figure 3). Both 0–10 mg/L Trichlorfon and Glyphosate promoted luminescence of *Photobaclerium phosphortum* T3, and with maximum luminous intensity at concentrations of 0.05 mg/L and 4 mg/L, respectively (Figure 4). In addition, 0–0.005 mg/L chromium and 0–0.008 mg/L lead also promoted the luminous intensity of *Photobaclerium phosphortum* T3 (Figure 5). The luminous intensity was also enhanced at concentrations of 0–0.002 mg/L Gallic acid and 0–0.005 mg/L Nonanoic acid, respectively (Figure 6). All 12 pollutants promoted the luminescence intensity of luminescent bacteria at low concentrations, and the promotion increased and then decreased.

All pollutants inhibited bacterial luminescence as the concentration continued to increase after a certain threshold concentration, and the rate of inhibition was positively correlated with the concentration of the chemical. When using luminescent bacteria to determine the toxicity of a chemical, a straight line is generally fitted to the inhibitory toxicity data to calculate the EC50 value. In this study, the correlation coefficients between the inhibition rate and the concentration of pollutants of the 12 selected pollutants (polycyclic aromatic hydrocarbons (PAHs), endocrine disruptors, antibiotics, pesticides, heavy metals, and plant metabolites) were high after they started to inhibit the luminescence intensity of the luminescent bacteria and the EC50 values were calculated in this way. The EC50 values of substances of the same species are also similar, and the toxicity of six types of substances to luminescent bacteria is as follows: endocrine disruptors > pesticides > antibiotics > heavy metals > polycyclic aromatic hydrocarbons > chemosensory agents (Table 1).

These results show that typical pollutants in water bodies do have a “Hormesis” effect on luminescent bacteria. Previous studies have also shown that sulfachloropyridazine (SCP) promotes the luminescence of *A. fischeri* ATCC7744 at low concentrations (9.2 × 10^−7^–2.5 × 10^−6^ mol/L); but by increasing concentrations of SCP, the luminescence of the bacteria showed inhibition [18]. Besides, similar phenomena were found in several substances such as sulfapyridine (SP), sulfamethoxazole (SMZ), sulfadiazine (SD), sulfisoxazole (SIZ), sulfamonomethoxine (SMM)sulfanilamide (SM), sulfamethoxazine (SMM), sulfadoxine (SDX), sulfapyridine (SPY), and sulfadiazine (SMR) [19]. Accumulating evidence has demonstrated that the hormesis of chemicals on organisms is highly generalizable, regardless of the biological model, endpoint measured, chemical class, and interindividual variability [24]. The effect of pollutants on luminescent bacteria is due to the interaction of the substance with the relevant proteins at low concentrations, leading to the accumulation of proteins and thus the stimulation of luminescence [18]. However, as the concentration of the substance increases and the concentration of the proteins exceeds a certain threshold, the increase in the concentration of the proteins may lead to inactivation of the proteins themselves [25,26]. Since hormesis has been increasingly recognized, the focus of research has gradually shifted from the high-dose effects of previously studied chemicals to a wider range of effects [27]. Therefore, there is an urgent need to characterize the luminous properties of luminescent bacteria under the influence of different substances.

### 3.2. Hormesis Parameters Based on the New Model

**Figure 7:**
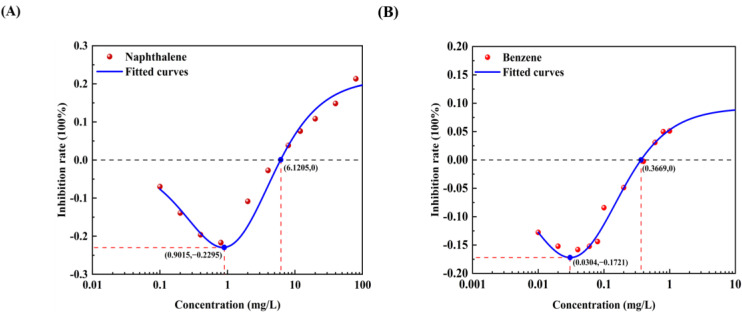
Fitted curves of inhibition of *P. phosphoreum* T3 luminescence by Naphthalene (**A**) and Benzene (**B**). The red dots indicate the rate of inhibition of *P. phosphoreum* T3 luminescence by pollutants at a given concentration. The blue dots with coordinates indicate the points of maximum enhancement and the enhancement–inhibition transition point.

**Figure 8:**
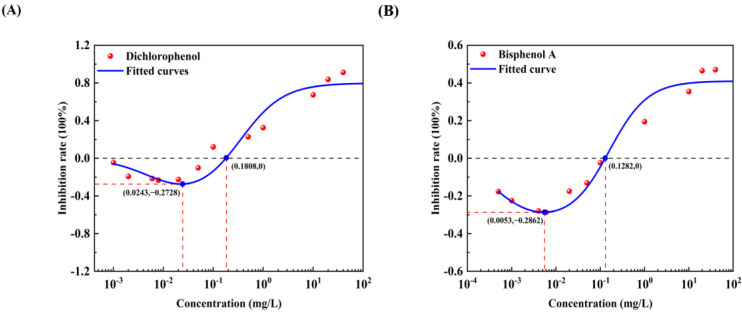
Fitted curves of inhibition of *P. phosphoreum* T3 luminescence by Dichlorophenol (**A**) and Bisphenol A (**B**). The red dots indicate the rate of inhibition of *P. phosphoreum* T3 luminescence by pollutants at a given concentration. The blue dots with coordinates indicate the points of maximum enhancement and the enhancement–inhibition transition point.

**Figure 9:**
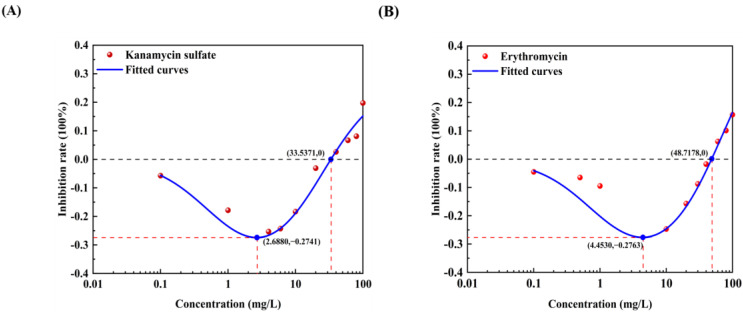
Fitted curves of inhibition of *P. phosphoreum* T3 luminescence by Kanamycin sulfate (**A**) and Erythromycin (**B**). The red dots indicate the rate of inhibition of *P. phosphoreum* T3 luminescence by pollutants at a given concentration. The blue dots with coordinates indicate the points of maximum enhancement and the enhancement–inhibition transition point.

**Figure 10:**
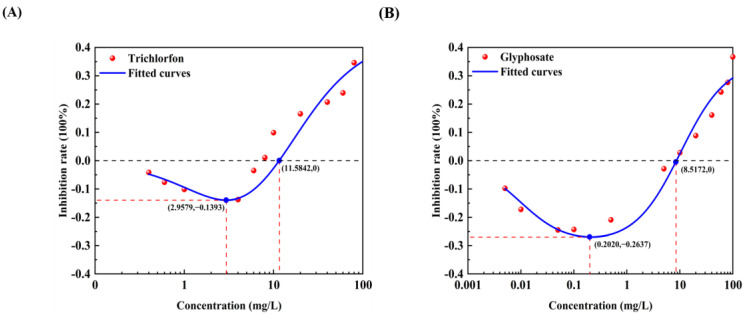
Fitted curves of inhibition of *P. phosphoreum* T3 luminescence by Trichlorfon (**A**) and Glyphosate (**B**). The red dots indicate the rate of inhibition of *P. phosphoreum* T3 luminescence by pollutants at a given concentration. The blue dots with coordinates indicate the points of maximum enhancement and the enhancement–inhibition transition point.

**Figure 11:**
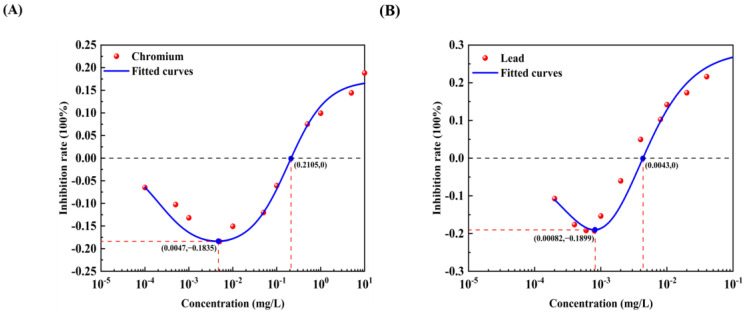
Fitted curves of inhibition of *P. phosphoreum* T3 luminescence by Chromium (**A**) and Lead (**B**). The red dots indicate the rate of inhibition of *P. phosphoreum* T3 luminescence by pollutants at a given concentration. The blue dots with coordinates indicate the points of maximum enhancement and the enhancement–inhibition transition point.

**Figure 12:**
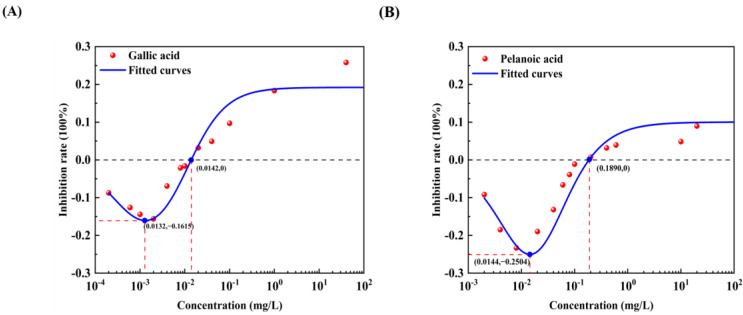
Fitted curves of inhibition of *P. phosphoreum* T3 luminescence by Gallic acid (**A**) and Nonanoic acid (**B**). The red dots indicate the rate of inhibition of *P. phosphoreum* T3 luminescence by pollutants at a given concentration. The blue dots with coordinates indicate the points of maximum enhancement and the enhancement–inhibition transition point.

The effect curves with 12 typical pollutants on the luminous intensity of *P. phosphoreum* T3 were obtained by fitting the obtained data points with the improved equation (Table 2). When the pollutant shows the promotion of luminescence intensity (the inhibition rate is negative), the promotion rate increases with the concentration of the substance and then decreases after reaching the maximum value (Table 3). When the concentration continued to increase, the luminous intensity of *P. phosphoreum* T3 was inhibited. The concentrations of different substances at which they promote luminescence in *P. phosphoreum* T3 to reach a maximum rate of promotion are substance dependent. Maximum promotions of 23% and 17% were obtained at concentrations of 0.902 mg/L and 0.030 mg/L for naphthalene and benzene, respectively (Figure 7). Dichlorophenol and bisphenol A showed the maximum promotions of luminescence intensity of 27% and 29% at 0.024 mg/L and 0.005 mg/L, respectively (Figure 8). For two antibiotics, kanamycin sulfate at 2.688 mg/L promoted luminescence intensity by a maximum of 27%, and erythromycin at 4.453 mg/L promoted luminescence intensity by a maximum of 28% (Figure 9). The maximum increase in luminescence intensity of bacteria was 14% and 26% under the influence of trichlorfon at 2.958 mg/L and glyphosate at 0.202 mg/L, respectively (Figure 10). The *P. phosphoreum* T3 was sensitive to both chromium and lead responses, with promotion reaching a maximum of 18% and 19% at concentrations of 0.005 mg/L and 0.001 mg/L, respectively (Figure 11). Gallic acid at 0.013 mg/L and nonanoic acid at 0.014 mg/L promoted luminescence by a maximum of 16% and 25%, respectively (Figure 12).

After the pollutant concentration increases to a certain threshold (Table 4), its promotion of bacterial luminescence is zero, and the pollutant inhibits bacterial luminescence as the concentration continues to increase. Concentrations of naphthalene and benzene greater than 6.121 mg/L and 0.367 mg/L, respectively, inhibited bacterial luminescence. Threshold concentrations of phenol and bisphenol A were 0.181 mg/L and 0.128 mg/L. 33.537 mg/L for kanamycin sulfate, respectively, and 48.718 mg/L for erythromycin also started to inhibit luminescence. Trichlorfon and glyphosate concentrations greater than 11.584 mg/L and 8.517 mg/L inhibited the luminescence of the bacteria. Concentrations of chromium greater than 0.211 mg/L and lead 0.004 mg/L showed negative effects on the bacteria. Gallic acid and nonanoic acid started to inhibit the luminescence of bacteria at 0.014 mg/L and 0.189 mg/L, respectively.

**Figure 13:**
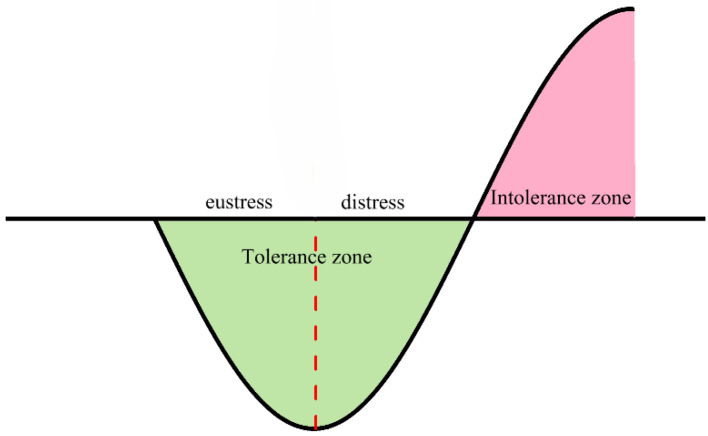
Pressure curves and zonation of pollutants on *P. phosphoreum* T3. The dotted line divides Tolerance zone into eustress and distress.

**Figure 14:**
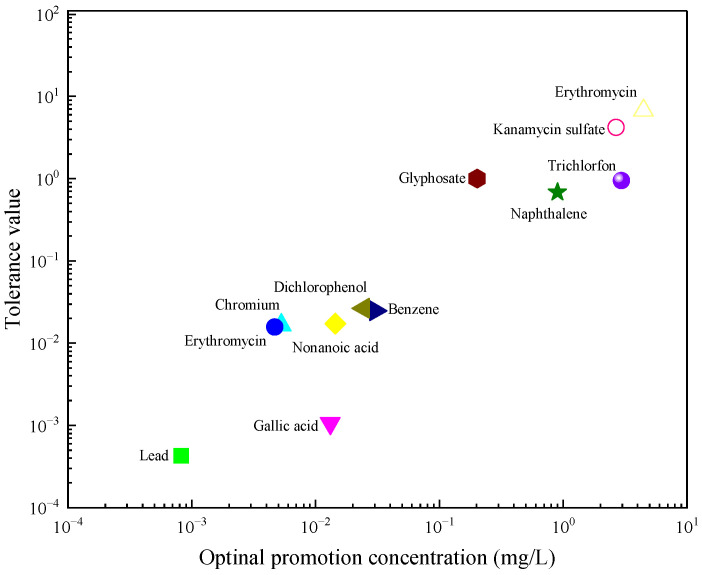
Optimal promoting concentration and tolerance of pollutants to *P. phosphoreum* T3 luminous intensity.

Based on the fitted effect curves, zones with negative inhibition rates were defined as tolerance zones, and regions with positive inhibition rates were defined as intolerance zones (Figure 13). Integration of the tolerance zone gives the degree of tolerance of the substance by *P. phosphoreum* T3 (Table 5). The higher the concentration at which the promotion rate is maximized, the greater the tolerance of the bacteria to the substance (Figure 14). Among them, the bacteria had the greatest values of tolerance to antibiotics (4.02 for kanamycin sulfate, 6.74 for erythromycin). The tolerance zone was further partitioned according to the characteristics of the change in inhibition rate. When the luminescent bacteria can completely adapt and form a new equilibrium after being pressurized, it is called an eustress zone; when the microalgae can only partially restore the equilibrium after being pressurized, it is called distress zone. The promotion of luminescence intensity by pollutants in the eustress zone gradually increases, and the promotion in the distress zone gradually decreases to 0. In the eustress zone, the substance always maintains a good facilitation effect; after reaching the optimal facilitation concentration, the facilitation effect is inhibited by a slight increase in concentration, which causes a rapid decrease in the facilitation effect (distress zone). The greater the concentration of substances in the non-tolerance zone, the greater the rate of inhibition of luminescence intensity, which further explains that when using the luminescent bacteria assay to determine the toxicity of a substance, it is important to do so in the range of concentrations at which the substance exhibits inhibition of the luminescent bacteria.

Numerous endeavors have been undertaken to integrate low-dose stimulatory effects into dose–response models, aiming to enhance their predictive accuracy and scientific rigor [28,29]. These attempts have been fueled by a growing recognition of the complexity of biological systems, where subtle changes in dosage can elicit non-monotonic responses, including stimulatory effects at lower concentrations that may be overshadowed by inhibitory effects at higher doses. Researchers have been successful in attaining this wholeness using a wide range of strategies to form these models. The traditional Weibull and logistic functions models were refined to be suitable for biphasic or hormetic patterns [19]. Sophisticated statistical approaches involving machine learning algorithms and non-linear regression methods are leveraged today to pinpoint and trace the exact extension of such environmentally health-promoting low-dose stimulatory effects. Through the integration of stimulatory effects at low doses in the models depicting the dose–response relationship, researchers hope to gain insights into the full spectrum of biological responses to various stimuli, thus contributing to the production of safer and more efficient therapeutics and chemicals, environmental health control, and risk assessment. Using this modeling analysis, the promotional properties of the contaminant for luminescent bacteria at low concentrations can be obtained and the maximum rate of promotion of luminescent intensity can be derived. In addition, when using luminescent bacteria to determine the toxicity of a contaminant, a threshold concentration can be used to determine the applicable concentration range, which is useful for obtaining more accurate and reliable toxicity data.

### 3.3. Hormesis Effect of Pollutants in Natural Waters on Luminescent Bacteria and Its Application

**Figure 15:**
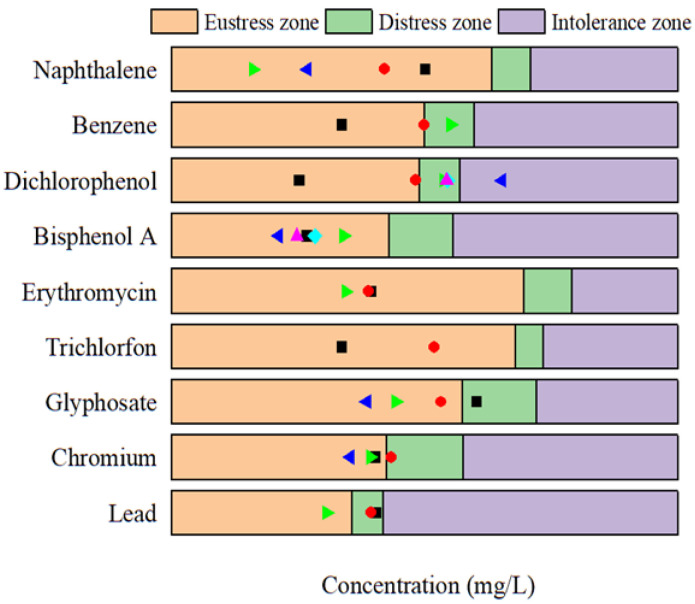
Concentration of pollutants in natural water. Dots of different shapes and colors indicate the concentration of the pollutant in the actual water.

The luminescent bacterial assay, renowned for its efficiency and convenience, has been frequently employed as a pivotal tool in assessing the toxicity of various substances. However, it is paramount to acknowledge that the toxicity of harmful substances often exhibits a concentration-dependent profile. The current characteristics of pollutants in actual water bodies are the presence of a wide variety of substances, with most at low concentrations (Figure 15). Nevertheless, with the continued utilization in production and daily life activities, these substances exhibit a slow but steady increase in concentration. Therefore, when utilizing bioluminescent bacteria assays for toxicity assessments of substances, it is crucial to consider whether they will have adverse effects on the bacteria at the target concentrations, ensuring accurate toxicity assessments amidst the dynamic nature of environmental pollution. Most of the 12 typical contaminants selected for this study are still in the tolerance zone of luminescent bacteria at current actual water concentrations. PAHs (0.004–0.032 mg/L), antibiotics (6.2 × 10^−7^–2.2 × 10^−6^ mg/L), pesticides (5.0 × 10^−7^–7.6 × 10^−6^ mg/L), and heavy metals (2.4 × 10^−7^–5.9 × 10^−6^ mg/L) promote luminescence in bacteria at current environmental concentrations, and the concentrations of these five groups of substances are in the positive stress zone, which means that the promotion of luminescence intensity will continue to increase and then decrease as these substances continue to accumulate in the water column. This means that using the luminescent bacteria method to determine the toxicity of these substances at actual water column concentrations is inaccurate. The concentrations of the two endocrine disruptors (2.2 × 10^−5^–1.548 mg/L) in water are relatively high and are already in the non-tolerance zone for bacteria in individual water bodies. This means that the use of a luminescent bacteria assay to determine the toxicity of endocrine disruptors at ambient concentrations is also not appropriate.

Most contaminants may promote the growth of luminescent bacteria at current environmental concentrations, so this method is not applicable to the detection of contaminants. However, the characterization of the effect of pollutants on luminescent bacteria at low concentrations can indeed be used for the detection and prediction of pollutant concentrations. Traditional methods for detecting pollutants, such as chromatography and spectrometry [30], focus solely on quantifying pollutant levels, rather than assessing their impact on organisms [31]. Therefore, in today’s era, the application of bioassays is one of the commonly adhered approaches by which individual or multiple pollutants are tested by measuring the responses biologically triggered by the specific organisms. Through the monitorization of luminescence intensity of the substance, it is possible not only to measure its concentration levels, but also to forecast concentration trends based on how the substance drives bacteria luminescence at low concentrations. This approach enables an initial assessment of how pollutants might affect aquatic organisms and human health at ambient concentrations. Pollutants do not exist singly in the environment, which makes it difficult to use luminescent bacteria to detect pollutants in natural waters. However, through the study of pure substances, the law of change of the influence of substances on luminescent bacteria can be derived, which can make up for the non-specificity of chemical substances on luminescent bacteria, making it possible to detect compound pollution.

## 4. Conclusions

Toxicity assessment is paramount in the effective management of pollutants, yet reliance solely on luminescent bacterial methods can yield misleading outcomes when quantifying the toxicity of contaminants. This research endeavor underscores this limitation by demonstrating that 12 representative pollutants exhibited a “Hormesis” effect on luminescent bacteria: enhancing luminescence at low concentrations but inhibiting it at higher levels. To comprehend this complex relationship, this study employed a modeling approach to fit the inhibition curves, incorporating parameters such as the optimal promoting concentration, maximum promoting rate, and tolerance threshold to dissect the underlying effects. Furthermore, this study reveals that the prevailing environmental concentrations of these pollutants tend to stimulate, rather than inhibit, bacterial luminescence. This revelation underscores the inadequacy of using this method in isolation for accurate toxicity evaluations. Nevertheless, the observed stimulatory effect of pollutants on bacterial luminescence presents a promising avenue for a novel approach to determining pollutant concentrations. By harnessing this promotional property, we can potentially develop more refined and accurate methods for monitoring and quantifying environmental pollutants. This study reveals the limitations of luminescent bacteria for the detection of substance toxicity by demonstrating complex concentration-dependent effects. This deepens our knowledge of biota–pollutant connections and is of utmost importance for research, monitoring progress, and environmental policies. It focuses on the role of science in decision-making. Furthermore, it enabled the development of new assessment methods that utilize the stimulatory properties of pollutants on bacterial luminescence as a potential tool for concentration determination.

## Figures and Tables

**Table 1:** EC50 values for pollutants.

Chemicals	EC50	R^2^
Naphthalene	19.07	0.97
Benzene	24.65	0.93
Dichlorophenol	10.40	0.95
Bisphenol A	11.87	0.82
Kanamycin sulfate	17.20	0.79
Erythromycin	19.29	0.98
Trichlorfon	14.76	0.97
Glyphosate	15.08	0.99
Chromium	20.22	0.98
Lead	19.00	0.99
Gallic acid	17.41	0.93
Nonanoic acid	37.16	0.88

**Table 2:** Parameters obtained under 12 pollutants by fitting the new dose–response model to *P. phosphoreum* T3.

Chemicals	Smax	Ks	Imax	Ki
Naphthalene	81.80	1.21	81.59	1.1899
Benzene	0.62	0.08	0.53	0.0173
Dichlorophenol	1.24	0.34	0.45	0.0061
Bisphenol A	0.75	0.16	0.34	0.0004
Kanamycin sulfate	0.71	23.24	0.43	0.6214
Erythromycin	0.94	68.04	0.40	0.8433
Trichlorfon	75.44	6.12	74.99	6.0167
Glyphosate	0.65	10.31	0.30	0.0100
Chromium	0.37	0.18	0.20	0.0002
Lead	71.61	0.00	71.33	0.0013
Gallic acid	0.46	0.01	0.27	0.0004
Nonanoic acid	143.77	0.02	143.67	0.0171

**Table 3:** Optimal promoting concentration and maximum promoting rate of *P. phosphoreum* T3 luminous intensity by pollutants.

Chemicals	Optimal Promotion Concentration (mg/L)	Optimal Promotion Concentration (mg/L)
Naphthalene	0.902	23
Benzene	0.030	17
Dichlorophenol	0.024	27
Bisphenol A	0.005	29
Kanamycin sulfate	2.688	279
Erythromycin	4.453	28
Trichlorfon	2.958	14
Glyphosate	0.202	26
Chromium	0.005	18
Lead	0.001	19
Gallic acid	0.013	16
Nonanoic acid	0.014	25

**Table 4:** Threshold concentrations for the effect of pollutants on *P. phosphoreum* T3 luminescence intensity.

Chemicals	Concentration at Effect Transition (mg/L)
Naphthalene	6.121
Benzene	0.367
Dichlorophenol	0.181
Bisphenol A	0.128
Kanamycin sulfate	33.537
Erythromycin	48.718
Trichlorfon	11.584
Glyphosate	8.517
Chromium	0.211
Lead	0.004
Gallic acid	0.014
Nonanoic acid	0.189

**Table 5:** Tolerance of pollutants by *P. phosphoreum* T3.

Chemicals	Tolerance Value
Naphthalene	0.6788
Benzene	0.0248
Dichlorophenol	0.0265
Bisphenol A	0.0167
Kanamycin sulfate	4.2023
Erythromycin	6.7410
Trichlorfon	0.9487
Glyphosate	1.0017
Chromium	0.0157
Lead	0.0004
Gallic acid	0.0011
Nonanoic acid	0.0173

## Data Availability

The original contributions presented in the study are included in the article, further inquiries can be directed to the corresponding author.
